# Pulsed Electric Field (PEF) and High-Power Ultrasound (HPU) in the Hurdle Concept for the Preservation of Antioxidant Bioactive Compounds of Strawberry Juice—A Chemometric Evaluation—Part I

**DOI:** 10.3390/foods12173172

**Published:** 2023-08-23

**Authors:** Anica Bebek Markovinović, Višnja Stulić, Predrag Putnik, Anamaria Birkić, Maja Jambrović, Dolores Šaško, Josipa Ljubičić, Branimir Pavlić, Zoran Herceg, Danijela Bursać Kovačević

**Affiliations:** 1 Faculty of Food Technology and Biotechnology, University of Zagreb, Pierottijeva 6, 10000 Zagreb, Croatia; anica.bebek.markovinovic@pbf.unizg.hr (A.B.M.); vstulic@pbf.hr (V.S.); anamaria.birkic@gmail.com (A.B.); maja.jambrovic@pbf.hr (M.J.); dsasko@pbf.hr (D.Š.); jljubicic@pbf.hr (J.L.); zherceg@pbf.hr (Z.H.); 2 Department of Food Technology, University North, Trg dr. Žarka Dolinara 1, 48000 Koprivnica, Croatia; 3 Faculty of Technology, University of Novi Sad, Blvd. Cara Lazara 1, 21000 Novi Sad, Serbia; bpavlic@uns.ac.rs

**Keywords:** hurdle technology, non-thermal technology, functional juice, polyphenolic content, storage

## Abstract

This work investigated the influence of pulsed electric field (PEF) and high-power ultrasound (HPU) combined with hurdle technology to preserve the bioactive compounds (BACs) content and antioxidant activity in stored strawberry juices. PEF was performed at 30 kV cm^−1^, 100 Hz during 1.5, 3, and 4.5 min, while HPU was performed at 25% amplitude and 50% pulse during 2.5, 5.0, and 7.5 min. Total phenols and hydroxycinnamic acids were the most stable BACs during the hurdle treatment without influence of the duration of both treatments, while flavonols and condensed tannins showed a significant stability dependence with respect to the duration of both treatments. Total phenols were also stable during storage, in contrast to the individual groups of BACs studied. A chemometric approach was used to optimize the parameters of the hurdle treatments with respect to the highest level of BACs and the antioxidant activity of the treated juices. In general, shorter treatment times in the hurdle approach resulted in better stability of BACs and antioxidant activity. The hurdle technology investigated in this study has the strong potential to be an excellent concept for optimizing the operating parameters of PEF and HPU technologies in the preservation of functional foods.

## 1. Introduction

Strawberry (*Fragaria* × *ananassa* Duch.) is a highly sensitive fruit and, therefore, has a short shelf life after harvest. Due to the rapid loss of quality, strawberries quickly lose value in the market [1]. To avoid potential losses and use high-quality raw material, ripe strawberry fruit should be processed appropriately as soon as possible. Strawberry products, just like fresh fruits, contain significant bioactive potential that can provide numerous health benefits, as shown by numerous studies on their antioxidant, anti-inflammatory, microbial modulatory, and cell-protective activities [2]. The functional properties of strawberry fruit are due to its rich composition of bioactive compounds (BACs). The bioactive potential of strawberries is mainly determined using a large number of different phenolic compounds, most of which have significant antioxidant activity [3]. 

Among polyphenols present in strawberries, flavonoids and their derivatives, including flavonols, flavonols, and anthocyanins, are the most abundant. After anthocyanins, flavonols dominate and can be present in both monomeric and polymeric forms called tannins [4,5]. Phenolic acids (e.g., derivatives of hydroxycinnamic acid and hydroxybenzoic acid) were also found in strawberries but in smaller amounts than flavonoids [4,6]. Since a large proportion of BACs transfer from the fresh fruit to the juice during processing, strawberry juice can be considered a functional food just like the fresh fruit [7].

In recent years, consumers are increasingly looking for highly nutritious and high-quality foods that are as close as possible to fresh and/or without chemical additives. Fruit juices are also becoming increasingly popular, mainly because they are consumed relatively quickly and easily and can replace a fresh fruit meal. Therefore, strawberry juice is increasingly in demand by consumers, not only for its appealing sensory properties but also for its nutritional and bioactive composition and health benefits, hence being very important for promoting human health and preventing diseases [8].

Juices are usually preserved using pasteurization because thermal processing ensures food safety and a longer shelf life. However, elevated temperatures during processing can decompose unstable BACs, such as polyphenolic compounds, thus reducing antioxidant activity, which ultimately affects the quality of the final product [9,10,11]. Accordingly, there has been an increased demand for technologies that not only preserve food but also do not affect its nutritional composition, are energy efficient, and can be used to reuse food industry by-products [12]. Therefore, advanced technologies in food production/processing, such as pulsed electric field (PEF) and high-power ultrasound (HPU), are increasingly becoming the focus of functional food processing [13]. 

The use of PEF technology has shown good results in maintaining good nutritional and sensory quality in treated fruit juices [14,15]. The use of HPU as a replacement for pasteurization is very promising, as the acoustic energy of ultrasound is directly transferred to the whole juice volume, significantly reducing the operating time. The higher energy savings compared to conventional methods validate this technology for industrial applications [16]. A recent trend in food preservation, known as the hurdle concept, involves the use of a carefully selected sequence of technologies, which can be either thermal and/or non-thermal, with the aim of preserving food quality and extending shelf life. The technologies used in this approach must be optimized in advance and are, therefore, typically operated under lower processing conditions than if they were used independently. In this way, potential negative effects of the technologies are avoided while their synergistic effects are promoted [17]. 

In juice processing, PEF and HPU technologies are often combined with other hurdles to maintain native quality characteristics [18,19,20,21]. To date, various combinations of hurdle technologies have been tested in the preservation of strawberry juices [22,23,24,25,26], but the combination of PEF and HPU as the most promising technologies for processing functional juices has not been investigated yet. Therefore, the aim of this study was to investigate the impact of PEF and HPU processing via the hurdle concept. In previous studies, the treatment parameters for both technologies were optimized [27,28] and adapted to the hurdle concept, so in this study, a further step was taken with the optimal parameters for PEF (30 kV cm^−1^, 100 Hz) and HPU (amplitude 25%, pulse 50%) selected. To better investigate the synergistic effect of these technologies when treated together, different treatment times were tested for PEF 1.5, 3.0, and 4.5 min and for HPU 2.5, 5.0, and 7.5 min. All strawberry juices were analyzed for their physicochemical properties, BAC content, and antioxidant activity before, immediately after treatment, and after 7 days of cold storage. The results were assessed using chemometric techniques to find and optimize the best combination of PEF and HPU technology for mutual strawberry juice treatment to best preserve the antioxidant bioactive compounds during cold storage.

## 2. Materials and Methods

### 2.1. Chemicals and Standards

HPLC 99% pure methanol was purchased from Honeywell (Paris, France). Folin-Ciocalteau (FC) reagent was obtained from Fisher Scientific UK (Loughborough, UK). Sodium carbonate, anhydrous (99.5–100.5%), sulfuric acid (96%, p.a.), hydrochloric acid (37%, *w*/*w*), and formic acid (98%, p.a.) were obtained from Lach-Ner (Neratovice, Czech Republic). Ethanol (96% pure) was purchased from Gram-mol (Zagreb, Croatia). Quercetin (95%) was purchased from Acros Organics (Guangzhou, China). Vanillin (99%) and chlorogenic acid (min. 95%) were obtained from Thermo Fisher (Kandel, Germany). DPPH (2,2-diphenyl-1-picrylhydrazyl radical), gallic acid standard (97.5–102.5%), and TPTZ (2,4,6-tris-2-pyridyl-*s*-triazine) were obtained from Sigma-Aldrich (St. Louis, MO, USA). Iron (III)-chloride hexahydrate and sodium acetate trihydrate resistant to potassium permanganate were obtained from Kemika (Zagreb, Croatia). Glacial acetic acid (≥99.8%) was purchased from Honeywell Fluka^TM^ (Seelze, Germany), and Trolox standard (6-hydroxy-2,5,7,8-tetramethylchroman-2-carboxylic acid) used for FRAP and DPPH assay was purchased from Biosynth (Bratislava, Slovakia).

### 2.2. Strawberry Juice Preparation

In 2022, strawberries (*Fragaria x ananassa* Duch, cultivar ‘Albion’) were grown at Jagodar HB in Donja Lomnica, Croatia, and picked at full maturity. The strawberries were taken to the laboratory after harvest. The stems were removed, and the fruits were washed with tap water, dried, and kept in hermetically sealed plastic containers at −18 °C until processing. A Kuvings B6000 slow juicer (VerVita d.o.o., Zagreb, Croatia) was used for juice processing using low-speed masticating technology (240 W, speed 60 rpm). The cloudy juices produced were immediately treated using the hurdle approach (Table 1).

### 2.3. Hurdle Concept consisting of Pulsed Electric Field (PEF) and High-Power Ultrasound (HPU) for Strawberry Juice Processing

**Figure 1:**
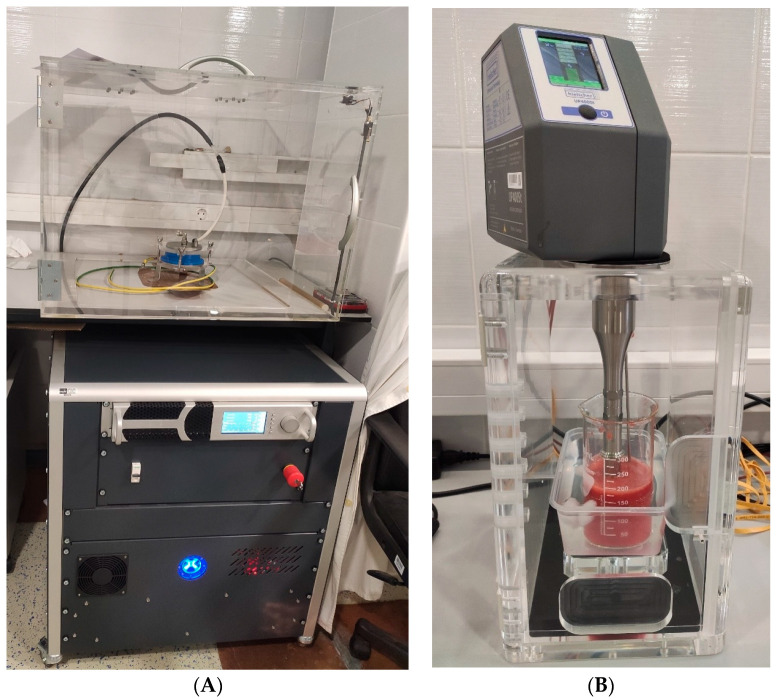
PEF device HVG60/1 PEF (Impel d.o.o., Zagreb, Croatia) (**A**) and ultrasonic processor Hielscher UP400St, 400 W, 24 Hz (Hielscher Ultrasonics GmbH, Teltow, Germany) (**B**).

Strawberry juice samples (200 mL) were first treated with PEF and then with HPU technology. The HVG60/1 HIPEF (Impel d.o.o., Zagreb, Croatia) instrument was used for PEF treatment (Figure 1A), and the Hielscher UP400St instrument (Hielscher Ultrasonics GmbH, Teltow, Germany) was used for HPU treatment (Figure 1B). 

The PEF device consisted of three units: a Magna control unit, a high-voltage power source, and a high-voltage pulse generator. The Magna control unit generates control pulses for the high-voltage system. The high-voltage power source converts the input voltage AC of 230 V into DC voltage in the range of 1 to 60 kV. The high-voltage pulse generator passes the input high voltage to the output in the form of pulses with specific parameters. Before starting the treatment, the device was set to the specified parameters. The samples were treated with 100 Hz, a voltage of 30 kV cm^−1^, a pulse width of 1 µs, and a duration of 1.5, 3.0, and 4.5 min (Table 1). The distance between the ground electrode and the high-voltage electrode was 2.5 cm. Both electrodes were made of stainless steel. During the PEF treatment, the temperature was measured with an infrared thermometer PCE-777 (PCE Instruments, Southampton, UK).

After PEF treatment, strawberry juice samples were subjected to HPU treatment with a titanium horn Ø 22 mm (Figure 1B). The UP400St device has a maximum power of 400 W, amplitude control from 20 to 100%, pulse control from 10 to 100%, and manually adjustable treatment duration. A digital thermometer (50 to 200 °C) was also incorporated into the apparatus for measuring the temperature of the sample prior to, during, and after treatment. Juice samples were treated with HPU parameters of amplitude 25%, pulse 50%, and duration time 2.5, 5.0, and 7.5 min. The control samples were untreated juices. During processing with PEF and HPU technologies, the temperature before treatment and the temperature after treatment were monitored. For PEF technology, the average initial temperature before the start of treatment was 17.16 °C, and after PEF treatment was 17.71 °C, so it can be assumed that there is no temperature difference. For HPU treatment, the average initial temperature before treatment was 16.61 °C, and after HPU treatment was 19.55 °C, so the influence of temperature can be neglected in this case.

All juice samples were stored in sterilized glass bottles that were securely sealed. After treatment with hurdle technology (PEF + HPU), one batch of juices was subjected to analysis instantly after processing, and another batch was subjected to storage at 4 °C for seven days. The quality indicators associated with physiochemical properties (pH and SSC), stability of BACs, and antioxidant activity were monitored in all juice samples. 

### 2.4. Determination of pH and Soluble Solids Content (SSC)

A Mettler Toledo FiveEasy pH meter (Mettler-Toledo GmbH, Greifensee, Switzerland) was used to measure pH, while a digital ATAGO Pal-3 refractometer (ATAGO Co., Tokyo, Japan), SSC (Brix%) was used to determine SSC. All measurements were performed in duplicates.

### 2.5. Extraction of Antioxidant Bioactive Compounds

Extraction of BACs from strawberry juice samples was performed using a modified method from the literature [7]. Briefly, 20 mL of 1% formic acid in 80% methanol (*v*/*v*) and 5 g of the sample were shaken for 1 min. The mixture was then extracted at 50 °C for 15 min in an ultrasonic bath (DT 514 H Sonorex Digitec 13.5 L, Bandelin electronic, Berlin, Germany). After filtration, the supernatant was diluted to 25 mL in a volumetric flask with extraction solvent. Until analysis, the extracts were stored at 18 °C in an inert gas environment.

### 2.6. Determination of Total Phenolic Content (TPC)

The modified Folin-Ciocalteau (FC) spectrophotometric assay described in the literature [29] was used to quantify the total phenolic content. 400 µL of the properly diluted extract, 400 µL of the FC reagent previously diluted fivefold with distilled water, and 4 mL of a 7.5% sodium carbonate solution (*w*/*v*) were added together. A spectrophotometer (LLG-uniSPEC 2 Spectrophotometer, Buch and Holm, Meckenheim, Germany) was used to measure the absorbance of the colored reaction at 725 nm after the reaction mixture had stood at room temperature for 20 min. Duplicate measurements were performed for each sample. Using a standard calibration curve created with various gallic acid concentrations (10–250 mg L^−1^), the TPC in the extracts was calculated. Results were expressed as mg gallic acid equivalent (GAE) per 100 g of sample.

### 2.7. Determination of Total Hydroxycinnamic Acids (HCA) and Total Flavonols (FL)

A modified spectrophotometric method was used to determine HCA and FL [30]. 250 µL of the extract was mixed with 250 µL of solution 1 (1 g L^−1^ HCl solution in 96% ethanol) and 4.55 mL of solution 2 (2 g L^−1^ HCl in distilled water). The reaction was shaken for 10 s and then continued for 30 min at room temperature and in the dark. Subsequently, a LLG-uniSPEC 2 Spectrophotometer (Lab Logistics Group GmbH, Meckenheim, Germany) was used to measure the color reaction at 320 nm for the measurement of HCA and the color reaction at 360 nm for the measurement of FL. The same steps were used to prepare the blank sample, but the extract was replaced by an extraction solvent. Parallel measurements were performed for each sample. The HCA content was determined using a calibration curve prepared with different concentrations of chlorogenic acid solution (10–600 mg L^−1^), while the amount of FL was determined with different concentrations of quercetin solution (10–600 mg L^−1^). The results of HCA content and FL were expressed as mg chlorogenic acid equivalent (CAE) per 100 g sample and mg quercetin equivalent (QE) per 100 g sample, respectively.

### 2.8. Determination of Condensed Tannins (CT)

The CT was calculated using a modified spectrophotometric technique that has been reported in the literature [31]. In summary, 2.5 mL of reagent 1 (25% H_2_SO_4_ solution in methanol) and 1 mL of extract were added to 2.5 mL of reagent 2 (a solution of 1% vanillin in methanol). The reaction mixture was homogenized for 1 min using a vortex shaker (Grant Instruments Ltd., Cambs, UK) and then left to stand at room temperature for 10 min. A LLG-uniSPEC 2 Spectrophotometer (Lab Logistics Group GmbH, Meckenheim, Germany) was then used to measure the color response at 500 nm. The same steps were used to create the blank sample, but the extract was substituted with an extraction solvent. For every sample, parallel measurements were made. Different quantities of catechin solution (10–120 mg L^–1^) were used to create a calibration curve, and the results were represented as mg of catechin equivalent (CA) per 100 g of the sample.

### 2.9. In Vitro Measurement of Antioxidant Activity

#### 2.9.1. 2,2-diphenyl-1-picrylhydrazyl Assay (DPPH)

The DPPH spectrophotometric technique described in the literature was used to evaluate the antioxidant activity [32]. In brief, 3 mL of a methanolic 0.5 mM DPPH solution and 1.5 mL of the extract were mixed. At room temperature and in the dark, the reaction mixture was allowed to react for 20 min. 1.5 mL of 100% methanol and 3 mL of a 0.5 mM DPPH solution were mixed as a control. Methanol was used as a blank. The absorbance of the color reaction was then measured at 517 nm. Parallel measurements were performed for each sample. Antioxidant activity was calculated using a calibration curve created using different concentrations of Trolox solution (10–150 µM), and results were expressed as µM Trolox equivalents (TE) per 100 g of sample.

#### 2.9.2. Ferric Reducing Antioxidant Power Assay (FRAP)

The spectrophotometric FRAP approach described in the literature was used to evaluate antioxidant activity [33]. 50 mL of acetate buffer (0.3 M) with a pH 3.6, 5 mL of a 10 mM tripyridyltriazine (TPTZ) solution prepared with HCl (40 mM), and 5 mL of a ferric chloride (FeCl_3_) solution (20 mM) were combined to prepare the FRAP reagent. Briefly, 4.5 mL of the FRAP reagent and 600 µL of the previously properly diluted extract were added to the glass tubes. Then, the reaction mixture was homogenized on a vortex shaker (Grant Instruments Ltd., Cambridge, UK) for 1 min, then thermostated in a water bath at 37 °C for 10 min. Subsequently, the absorbance of the reaction was determined at 593 nm using an LLG-uniSPEC 2 Spectrophotometer (Lab Logistics Group GmbH, Meckenheim, Germany). To prepare the blank sample, the same steps were followed, but the extract was replaced by an extraction solvent. A calibration curve was prepared using different concentrations of Trolox solution (10–150 µM). Results were presented as µM Trolox equivalents (TE) per 100 g of sample.

### 2.10. Statistical Analysis

Experiments were designed as full factorial randomized experimental designs (n = 40) (Table 1). Dependent variables were the contents of (i) total phenols (TPC; mg 100 g^−1^); (ii) hydroxycinnamic acids (HCA; mg 100 g^−1^); (iii) flavonols (FL; mg 100 g^−1^); (iv) condensed tannins (CT; mg 100 g^−1^); (v) DPPH assay (mg 100 g^−1^); and (vi) FRAP assay (mg 100 g^−1^). Independent variables were: (i) exposure to PEF (1.5, 3.0, and 4.5 min); (ii) exposure to HPU (2.5, 5.0, and 7.5 min); and (iii) duration of storage (0 and 7 days). Descriptive statistics were used to assess the basic information on the experimental data set. Differences between treatments (continuous variables) were tested using multivariate analysis of variance (MANOVA). The Pearson coefficient was used to assess correlations between pairs of continuous variables. Exploratory hierarchical Ward’s cluster analysis was used to measure standardized similarities in samples. In nonparametric analysis, the Kruskal–Wallis test was employed. The significance levels for rejection of a null hypothesis were α ≤ 0.05 in all tests. Analyses were performed using IBM SPSS Statistics (v.24), and experimental design was performed using Statgraphics Centurion^®^ (StatPoint Technologies, Inc., Warrenton, VA, USA).

## 3. Results and Discussion

### 3.1. Use of Chemometrics to Evaluate Hurdle Processed Samples vs. Control Samples

**Figure 2:**
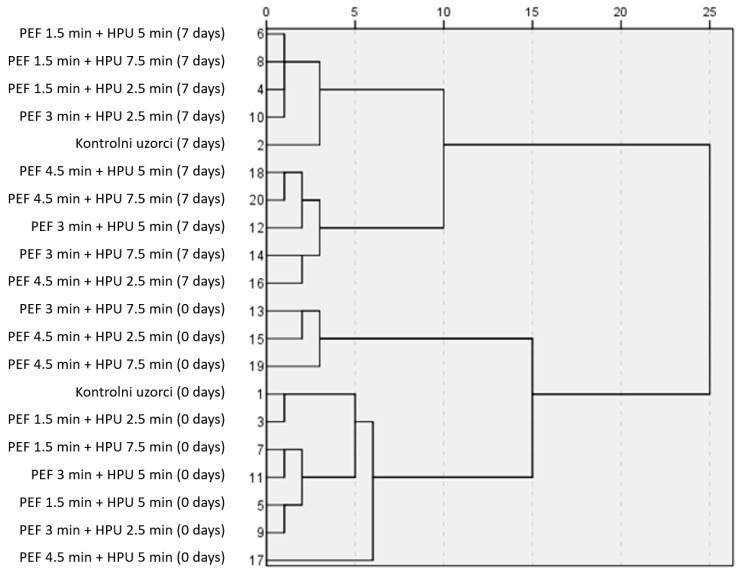
Results of the hierarchical clustering for averaged and standardized juice samples. PEF—Pulsed electric field, HPU—High power ultrasound.

Chemometric evaluation of data from hurdle-processed samples compared to control (untreated) samples was conducted to investigate the impact of hurdle technology on strawberry juice quality. Ward’s hierarchical clustering method showed that in the case where all juice samples were considered for standardized similarities, PEF-treated samples were most similar to controls with 1.5 min and 2.5 min with HPU at the beginning of the storage (0 day). On the 7th day of storage, samples treated with PEF for 1.5 min and HPU for 2.5–7.5 min were most similar to controls. Other samples that were similar to controls were treated with PEF for 3 min and HPU for 2.5 min (Figure 2). 

Lower times of both treatments favored the preservation of BACs and antioxidant activity, making the treated samples most similar to the control samples. Previously, it was found that anthocyanin content was largely dependent on the strength and duration of PEF treatment [34]. Thus, it is reasonable to assume that a shorter treatment duration for both technologies favors the preservation of native polyphenol content as found in the untreated samples (i.e., controls). Next, the authors reported the same trend in DPPH antioxidant activity, with values decreasing with increasing HIPEF treatment duration [35]. That control samples of strawberry juices were most similar to HPU-treated samples was previously confirmed for the shortest treatment durations [24,35]. Nevertheless, studies have also shown that longer HPU treatment resulted in better yield of TPC and increased antioxidant activity, such that the longer treated samples had higher levels of BAC and antioxidant activity than the shorter treated samples, while the least treated samples had the most similar levels of BAC and antioxidant activity with the control samples [24,35]. This is because the stability of BACs is cumulatively and synergistically affected by other process parameters of the applied technologies [36].

**Figure 3:**
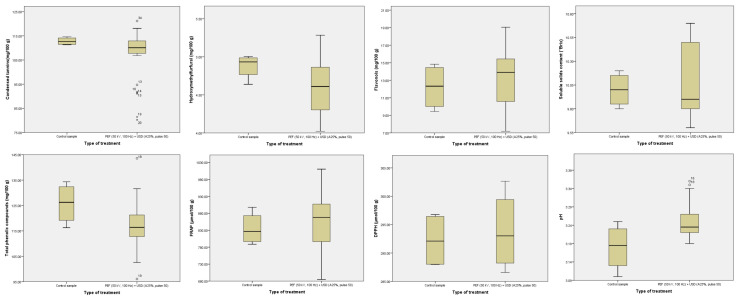
Median values of BAC content, antioxidant activity, SSC, and pH in control vs. hurdle-treated samples. All data are presented as median (25th–75th percentile).

Overall, the control samples did not differ from the hurdle-treated samples in terms of the BACs assayed, antioxidant activity, and SSC (Figure 3). The only difference was the median pH, where untreated samples were more acidic than the hurdle samples. Although our results showed a statistically significant change in pH, no changes in pH were detected in the spinach juice samples, neither for the untreated sample nor for the HPU-treated (40 kHz, 200 W, 21 min), the PEF-treated (9 kV cm^−1^, 1 kHz, 60 mL min^−1^, 30 ± 2 °C, time: 335 μs), and for the hurdle processed (HPU + PEF) [37]. However, Makroo et al. [38] observed a significant increase in pH after 15 s of ohmic heating of watermelon juice. This could be due to the fact that electrolysis or electrochemical interactions occurred during the treatment [39]. Table 2 shows the numerical values of the Kruskal–Wallis test of the hurdle-treated samples compared to untreated samples, which correlates with the graphical representation in Figure 3. 

### 3.2. The Changes in BACs, Antioxidant Activity, SSC, and pH in Strawberry Juices after Hurdle Technology Processing and Storage

Before processing, the operating parameters need to be optimized to meet the high-quality requirements of the processed food. The results for the influence of hurdle parameters and storage on BAC, antioxidant activity, SSC, and pH of strawberry juices are presented in Table 3. Strawberry juices were found to be a good source of BACs, namely a TPC value of 117.93 ± 0.70 mg 100 g^−1^. CT was the predominant bioactive compound detected (107.66 ± 0.23 mg 100 g^−1^), followed by HCA (28.03 ± 0.28 mg 100 g^−1^) and FL (13.38 ± 0.19 mg 100 g^−1^). During storage, a TPC value of 117.93 ± 0.70 mg 100 g^−1^ was observed, and this value remained constant during the experimental period. The SSC value was also constant during storage, with an average value of 10.12 ± 0.01 °Brix. The results obtained are similar to those of Sulaiman et al. [40], where the SSC value remained constant in strawberry puree samples treated with ultrasound (24 kHz, 1.3 W g^−1^, 33 °C) and stored at 3 °C for 30 days. 

However, HCA and FL content decreased by 28% and 12%, respectively, during storage, as did DPPH and FRAP, which decreased by 4% and 3%, respectively, during the same period. The same trend was observed for pH but in a much lower range. Interestingly, the value of CT increased by 7%, perhaps implying that the losses of HCA and FL could be related to condensation to larger tannin molecules. This is also consistent with the observation that the amount of TPC remained constant since there was no real overall loss of polyphenols, as they probably only changed their form due to the condensation process. In addition, the results obtained are partly in accordance with our earlier work, in which HIPEF-treated strawberry juice samples exhibited a decrease in FL levels and an increase in CT levels during 7 days of cold storage [28].

When observing only PEF processing in the hurdle treatment, it can be seen that the total amount of TPC remained stable, regardless of how long the samples were exposed to PEF. These results are consistent with previously published work in which strawberry juices treated with HIPEF (40 and 50 kV cm^−1^, 100 and 200 Hz) had no statistical effect on the changes in TPC amounts as a function of treatment duration of 3 and 6 min [28]. Of all the polyphenols studied, prolonged PEF treatment increased only HCA by 7% and DPPH by 6%. Similarly, for onion extracts, a positive correlation between antioxidant activity and electric field strength and duration of PEF treatment was found [41].

SSC and pH followed the same direction as the previously mentioned compounds but with a lower percentage (1%) at the lowest and the most intense exposure. Other polyphenols generally decreased with increasing duration of PEF treatment. Between 1.5 and 4.5 min of PEF, the percentage of FL and CT decreased by approximately 10% and 8%, respectively. Similarly, increasing PEF treatment duration from 3 to 6 minutes resulted in a statistically significant decrease in FL and CT [28]. Interestingly, FRAP followed a parabolic trend in which the highest value was observed at intermediate intensity and then fell to the initial level at the longest PEF treatment. 

Independently, HPU did not affect HCA and pH, but the most intense exposure to this treatment decreased the levels of TPC, FL, CT, and FRAP. HPU treatments beyond 5 min decreased the content of TPC by 4%, FL by 10%, CT by 7%, and FRAP decreased by 6%. In a previous study with apple juice, HPU decreased the content of TPC by 32.94% FL by 21.66%, while the antioxidant activity decreased by 23.76% (DPPH) and 27.49% (FRAP) [42], suggesting that HPU in combination with other hurdles is less invasive to sensitive BACs in fruit juices. In HPU-processed strawberry juice, the processing time had no significant influence on the content of HCA, whereas the content of FL and CT was reduced by increasing the treatment duration from 5 to 10 min [27]. The observed trend is consistent with the obtained results, as the content of FL and CT increased when the treatment duration was increased from 5 to 7.5 min. In a study by Adekunte et al. [43], tomato juice was treated with HPU (20 kHz, 24.4–61.0 µm, 2–10 min), and the different operating times did not affect the changes in pH and SSC of the juice.

In our case, increasing the operating time from 2.5 to 5 min did not change the SSC value. However, when extended further to 7.5 min, an increase was observed. This could have a positive effect on stability during shelf-life, as a higher SSC value could inhibit the decrease in ascorbic acid and thus have a positive effect against oxidation [44].

When observing the impact of HPU on the content of bioactive antioxidants, a processing time of 5 min resulted in the highest content of TPC, FL, CT, and antioxidant activity (FRAP). Antioxidant bioactive compounds in the vacuole may be in soluble form or covalently bound to the cell wall matrix. Therefore, it is possible that the use of HPU favored the destruction of cell wall material and made it easier to release their constituents by cavitation collapsing around the colloidal particles [45]. A further extension to 5 min resulted in a reduction in these compounds. It is possible that this is a disintegration of the bioactive antioxidant due to long-term HPU treatment, which may cause excessive cavitation and cell breakup of the product [45]. DPPH was the only parameter that increased by almost half a percent after 5 min of HPU treatment, which may be due to the fact that it is related to the chemical changes and enhanced recovery of BACs. 

To examine the effects of the hurdle technique, i.e., first combining the least exposure to PEF with all exposures to HPU, the following observations were made. Here, 1.5 min of PEF combined with 2.5, 5.0, and 7.5 min of HPU had no effect on TPC, HCA, FRAP, and pH. SSC and the levels of the other bioactive compounds decreased either linearly or parabolically with increasing HPU exposure, whereas DPPH was the only parameter that actually increased linearly with HPU exposure.

When the experiment was repeated with the same HPU settings but with an extension of PEF exposure to 3 min, the following was observed. Still, no changes were observed in the levels of TPC, HCA, FRAP, and PH. However, new patterns appeared in the data. Thus, FL and DPPH showed U-shaped curves; CT decreased linearly at 5%, while SSC increased linearly at 7% after 5 min exposure to HPU. To date, no studies have been performed on the influence of the treatment duration of the combination of PEF and HPU technology on the BACs and antioxidant capacities and physicochemical properties studied in this work.

The last part of the experiment included the highest PEF load (4.5 min) in combination with all previous HPU settings (2.5–7.5 min). Here, as before, no changes were observed in HCA, FRAP, and pH variables, but DPPH was added to this group of variables, while TPC showed significant changes in this hurdle setting. TPC, FL, and CT showed parabolic trends, meaning that the longest PEF exposure best matched with the mean exposure (5 min) to HPU. A linear trend was observed only for SSC, where the longest PEF/HPU exposure decreased the level of SSC by 3%. These data strongly suggest that the longest exposure to hurdle treatment causes the cellular material in the samples to degrade and release polyphenols in the matrix until they are depleted, whereupon the mechanical forces of the hurdle technology (possibly HPU) begin to degrade them back to their original levels (or further). These data strongly suggest that optimizations are needed to identify “inflexion points” and ensure the highest possible nutritional value of the samples (in terms of polyphenolic compounds).

### 3.3. Optimization of Operating Parameters of Hurdle Technology for Strawberry Juice Treatments

Table 4 shows the mutual correlations between hurdle technology parameters and BAC content, antioxidant activity, SSC, and pH. Overall, prolonged storage decreased HCA, DPPH, and pH. El Darra et al. [46] recorded a decrease in pH when red grape extracts were stored during a 30-day of alcoholic fermentation. Moreover, prolonged exposure to PEF favored the increase in SSC and pH and the decrease in CT, while exposure to HPU showed no general direction. These results are in agreement with previous studies by Bebek Markovinović et al. [28], in which prolonged exposure of strawberry juice to HIPEF treatment from 3 to 6 min caused a statistically significant decrease in CT. TPC was positively associated with CT and FRAP, as this group of compounds was the most abundant in the samples and probably responsible for the antioxidant activity. With increasing HCA content, the content of FL increased, while the content of CT was reduced. This could be due to the fact that HCA condenses to tannins. In addition, HCA was strongly positively associated with DPPH, indicating that they contribute most to the antioxidant activity of the samples, and strongly negatively associated with decreasing pH, which makes sense since they contribute to the acidity of the samples. As the content of CT increased, DPPH and pH decreased, while FRAP increased. 

**Figure 4:**
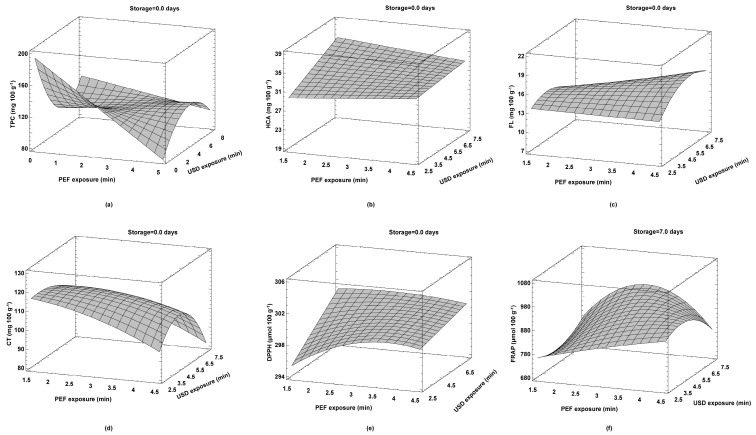
Optimal hurdle (PEF/HPU) treatment for maximum content of BACs and antioxidant activity: (**a**) total phenolic content (mg 100 g^−1^); (**b**) hydroxycinnamic acids (mg 100 g^−1^); (**c**) flavonols (mg 100 g^−1^); (**d**) condensed tannins (mg 100 g^−1^); (**e**) DPPH assay (µmol 100 g^−1^); (**f**) FRAP assay (µmol 100 g^−1^).

In order to obtain a product with preserved nutritional value, i.e., with a maximum content of native BACs, the operating parameters of the hurdle technology must be optimized. An optimal TPC of 125.81 mg 100 g^−1^ was obtained with a hurdle combination of 1.5 min PEF treatment and 2.5 min HPU treatment. Similarly, the highest CT content of 116.72 mg 100 g^−1^ was obtained with 1.5 min PEF and 3.2 min HPU treatment (Table 5, Figure 4). The best recovery of TPC and CT in strawberry juices was favored by shorter treatment time (4 min and 4.7 min of total treatment, respectively). Moreover, in this case, CT is one of the most abundant compounds observed in strawberry juices, and due to its complex macromolecular structure, it is possible that a longer treatment time after the initial desirable extraction by the cavitation effect subsequently causes its destabilization and disintegration. Therefore, it is possible that CT and TPC correspond to a shorter treatment time, during which the destabilization of the cellular structure of the fruit tissue was achieved. This could facilitate the extraction of these compounds during the electroporation and cavitation process, but further exposure to these technologies would lead to their destabilization and destruction [47,48,49].

Similarly, the same PEF, but with a longer HPU exposure of 7.5 min, resulted in a maximum HCA content of 35.56 mg 100 g^−1^. Longer exposure to both PEF (4.5 min) and HPU (5.8 min) promoted an increased FL content, which was 17.36 mg 100 g^−1^. In contrast to the recovery times of TPC and CT, the best yield of HCA and FL was obtained in longer hurdle treatments (9 min and 10.3 min, respectively). Koraqi et al. [50] showed that a longer time of ultrasound-assisted extraction increased the yield of phenolic and flavonoid compounds in relation to antioxidant activity values. At similar treatment times, 10.2 min and 10.5 min, the highest values of antioxidant capacities, DPPH and FRAP, were obtained. It seems that the best antioxidant activity in the samples was obtained at shorter exposure to PEF (2.7 min for DPPH; 3.0 min for FRAP) and longer exposure to HPU (both assays reached the maximum at 7.5 min). One of the possible explanations could be the fact that both components, HCA and FL, correlated positively with DPPH values (Table 3), and this could explain why it took a long time for complete extraction. As expected, all polyphenols show the highest values at the beginning of storage (0 days). These results are in contrast with previously published work, in which optimization of the parameters of the HIPEF technology (40 and 50 kV cm^−1^, frequency 100 and 200 Hz, and treatment duration 3 and 6 min) showed that storage for 7 days at 4 °C favored the highest yield of TPC, HCA, and CT in strawberry juices [28]. This inconsistency could indicate a subsequent positive effect of the HIPEF technology, which caused easier straining of the extracted studied compounds during storage, which did not happen in our case because milder treatment conditions, i.e., hurdle technology, were applied. An interesting difference between the antioxidant assays relates to the duration of storage, where DPPH was highest at the beginning of storage, while FRAP peaked at the end of storage. This may be related to the subsequent extraction of BACs, which were not studied in this work and may have contributed in some way to the increased antioxidant activity during storage specifically demonstrated one antioxidant assay over the other.

## 4. Conclusions

Recently, there has been an increased search for sustainable processing for efficient preservation. In this regard, the hurdle concept is being explored, which combines the use of multiple hurdles (e.g., advanced thermal/non-thermal technology) with the goal of preserving the quality of functional foods. The results of this research confirm that PEF and HPU, in combination with chemometric optimization of the results, can be a good alternative to conventional preservation in strawberry fruit juice processing. Among the investigated BACs in strawberry juices, total phenols and hydroxycinnamic acids showed the highest stability when treated with hurdle technology, while total phenols were also the most stable during storage compared to the other individual groups of BACs. Considering physicochemical properties, BAC content, and antioxidant activity, the strawberry juice samples treated with a combination of PEF of 1.5 min and HPU of 2.5 min were most similar to the untreated samples stored for 0 days. The samples that were most similar to the controls after 7 days of storage were those treated with a combination of 1.5 min PEF and 2.5–7.5 min HPU. These results suggest that the combination of both technologies has a positive effect on maintaining the quality of strawberry juices, both after treatment and during cold chain storage.

By optimizing the hurdle technology parameters, the highest yield of BACs and antioxidant activity was obtained at 0 days of storage, except for the FRAP value, which was highest at 7 days of storage. The highest yield of TPC was obtained when treated with PEF (1.5 min) and HPU (2.5 min) with 125.81 mg 100 g^−1^. This was followed by condensed tannins with 116.72 mg 100 g^−1^ when treated with PEF (1.5 min) and HPU (3.2 min), hydroxycinnamic acids when treated with PEF (1.5 min) and HPU (7.5 min), and finally flavonols when treated with PEF (4.5 min) and HPU (5.8 min). The highest DPPH values were measured in treatment with PEF (2.7 min) and HPU (7.5 min) and FRAP in treatment with PEF (3.0 min) and HPU (7.5 min).

It can be concluded that PEF and HPU in a hurdle combination can be considered for broader applications as two innovative technologies that complement each other thanks to their different mechanisms of action (e.g., electroporation, cavitation) and have a mutually beneficial synergistic effect on the stability of BACs. Optimization performed with chemometric tools has shown that shorter treatment times are more favorable for maintaining the biological value of juices. However, this technology should be further tested for the stability of other quality parameters, which would confirm this concept as promising for the juice processing industry.

## Figures and Tables

**Table 1:** An experimental research design.

Sample of Juice	Storage (Days)	Processing	PEF Exposure (min)	HPU Exposure (min)
1	0	Control	/	/
2	0	PEF + HPU	1.5	2.5
3	0	PEF + HPU	1.5	5
4	0	PEF + HPU	1.5	7.5
5	0	PEF + HPU	3	2.5
6	0	PEF + HPU	3	5
7	0	PEF + HPU	3	7.5
8	0	PEF + HPU	4.5	2.5
9	0	PEF + HPU	4.5	5
10	0	PEF + HPU	4.5	7.5
11	7	Control	/	/
12	7	PEF + HPU	1.5	2.5
13	7	PEF + HPU	1.5	5
14	7	PEF + HPU	1.5	7.5
15	7	PEF + HPU	3	2.5
16	7	PEF + HPU	3	5
17	7	PEF + HPU	3	7.5
18	7	PEF + HPU	4.5	2.5
19	7	PEF + HPU	4.5	5
20	7	PEF + HPU	4.5	7.5

Control—untreated strawberry juice; PEF + HPU—strawberry juice treated with PEF nad HPU technology in hurdle concept.

**Table 2:** Results for Kruskal–Wallis test (hurdle treatment vs. control samples).

	TPC	HCA	FL	CT	DPPH	FRAP	SSC	pH
Chi-Square	3.417	1.829	0.52	2.35	0.813	0.587	0.134	4.64
df	1	1	1	1	1	1	1	1
Sig.	0.07	0.18	0.47	0.13	0.37	0.44	0.71	0.03 *

* Kruskal–Wallis test is significant at the *p* ≤ 0.05. TPC—total phenolic content (mg 100 g^−1^); HCA—hydroxycinnamic acids (mg 100 g^−1^); FL—flavonols (mg 100 g^−1^); CT—condensed tannins (mg 100 g^−1^); Antioxidant activity—DPPH (µmol 100 g^−1^) and FRAP (µmol 100 g^−1^); SSC—soluble solids content (°Brix).

**Table 3:** Effect of hurdle technology on bioactive compounds content, antioxidant activity, SSC, and pH of strawberry juices during storage.

	n	TPC	HCA	FL	CT	DPPH	FRAP	SSC	pH
Storage		*p* = 0.39 ^‡^	*p* ≤ 0.01 ^†^	*p* ≤ 0.01 ^†^	*p* ≤ 0.01 ^†^	*p* ≤ 0.01 ^†^	*p* = 0.02 ^†^	*p* = 0.26 ^‡^	*p* ≤ 0.01 ^†^
0 days	18	118.54 ± 0.99 ^a^	32.58 ± 0.40 ^a^	14.26 ± 0.27 ^a^	104.15 ± 0.33 ^b^	299.30 ± 0.13 ^a^	866.54 ± 7.66 ^a^	10.11 ± 0.01 ^a^	3.28 ± 0.01 ^a^
7 days	18	117.31 ± 0.99 ^a^	23.49 ± 0.40 ^b^	12.50 ± 0.27 ^b^	111.18 ± 0.33 ^a^	288.57 ± 0.13 ^b^	838.29 ± 7.66 ^b^	10.13 ± 0.01 ^a^	3.22 ± 0.01 ^b^
PEF		*p* = 0.48 ^‡^	*p* = 0.02 ^†^	*p* ≤ 0.01 ^†^	*p* ≤ 0.01 ^†^	*p* ≤ 0.01 ^†^	*p* ≤ 0.01 ^†^	*p* ≤ 0.01 ^†^	*p* = 0.02 ^†^
1.5 min	12	118.19 ± 1.22 ^a^	26.79 ± 0.49 ^b^	14.15 ± 0.33 ^a^	110.96 ± 0.40 ^a^	292.77 ± 0.15 ^b^	830.50 ± 9.38 ^b^	9.85 ± 0.02 ^c^	3.23 ± 0.01 ^b^
3 min	12	118.85 ± 1.22 ^a^	28.86 ± 0.49 ^a^	13.57 ± 0.33 ^a^	109.94 ± 0.40 ^a^	294.69 ± 0.15 ^a^	879.81 ± 9.38 ^a^	10.01 ± 0.02 ^b^	3.26 ± 0.01 ^a^
4.5 min	12	116.75 ± 1.22 ^a^	28.46 ± 0.49 ^a^	12.44 ± 0.33 ^b^	102.09 ± 0.40 ^b^	294.34 ± 0.15 ^a^	846.92 ± 9.38 ^b^	10.49 ± 0.02 ^a^	3.26 ± 0.01 ^a^
HPU		*p* = 0.02 ^†^	*p* = 0.63 ^‡^	*p* = 0.02 ^†^	*p* ≤ 0.01 ^†^	*p* ≤ 0.01 ^†^	*p* ≤ 0.01 ^†^	*p* ≤ 0.01 ^†^	*p* = 0.26 ^‡^
2.5 min	12	118.51 ± 1.22 ^a,b^	28.42 ± 0.49 ^a^	13.31 ± 0.33 ^a,b^	107.96 ± 0.40 ^b^	293.54 ± 0.15 ^b^	854.81 ± 9.38 ^a^	10.11 ± 0.02 ^b^	3.25 ± 0.01 ^a^
5 min	12	120.24 ± 1.22 ^a^	27.84 ± 0.49 ^a^	14.14 ± 0.33 ^a^	111.46 ± 0.40 ^a^	293.62 ± 0.15 ^b^	877.46 ± 9.38 ^a^	10.07 ± 0.02 ^b^	3.25 ± 0.01 ^a^
7.5 min	12	115.04 ± 1.22 ^b^	27.84 ± 0.49 ^a^	12.70 ± 0.33 ^b^	103.57 ± 0.40 ^c^	294.64 ± 0.15 ^a^	824.96 ± 9.38 ^b^	10.18 ± 0.02 ^a^	3.26 ± 0.01 ^a^
PEF + HPU (hurdle)		*p* = 0.07 ^‡^	*p* = 0.59 ^‡^	*p* = 0.03 ^†^	*p* ≤ 0.01 ^†^	*p* ≤ 0.01 ^†^	*p* =0.78 ^‡^	*p* = 0.03 ^†^	*p* = 0.76 ^‡^
1.5 min + 2.5 min	12	122.79 ± 1.90 ^a^	26.14 ± 0.84 ^a^	12.80 ± 0.76 ^b^	113.62 ± 0.52 ^a^	290.94 ± 0.22 ^c^	840.84 ± 17.48 ^a^	9.93 ± 0.04 ^a^	3.23 ± 0.01 ^a^
1.5 min + 5 min	12	115.91 ± 1.90 ^a^	26.81 ± 0.84 ^a^	16.36 ± 0.76 ^a^	110.47 ± 0.52 ^b^	293.09 ± 0.22 ^b^	825.53 ± 17.48 ^a^	9.88 ± 0.04 ^a^	3.23 ± 0.01 ^a^
1.5 min + 7.5 min	12	115.86 ± 1.90 ^a^	27.41 ± 0.84 ^a^	13.29 ± 0.76 ^b^	108.78 ± 0.52 ^b^	294.30 ± 0.22 ^a^	825.14 ± 17.48 ^a^	9.75 ± 0.04 ^b^	3.23 ± 0.01 ^a^
PEF + HPU (hurdle)		*p* = 0.39 ^‡^	*p* = 0.25 ^‡^	*p* ≤ 0.01 ^†^	*p* ≤ 0.01 ^†^	*p* ≤ 0.01 ^†^	*p* = 0.09 ^‡^	*p* ≤ 0.01 ^†^	*p* = 0.76 ^‡^
3 min + 2.5 min	12	118.18 ± 2.15 ^a^	29.23 ± 0.82 ^a^	15.19 ± 0.41 ^a^	110.25 ± 0.90 ^a^	294.94 ± 0.25 ^a^	836.0 5± 19.66 ^a^	9.75 ± 0.03 ^b^	3.25 ± 0.01 ^a^
3 min + 5 min	12	116.99 ± 2.15 ^a^	27.63 ± 0.82 ^a^	11.95 ± 0.41 ^c^	113.32 ± 0.90 ^a^	293.64 ± 0.25 ^b^	895.61 ± 19.66 ^a^	9.80 ± 0.03 ^b^	3.25 ± 0.01 ^a^
3 min + 7.5 min	12	121.37 ± 2.15 ^a^	29.72 ± 0.82 ^a^	13.55 ± 0.41 ^b^	106.25 ± 0.90 ^b^	295.48 ± 0.25 ^a^	907.78 ± 19.66 ^a^	10.48 ± 0.03 ^a^	3.28 ± 0.01 ^a^
PEF + HPU (hurdle)		*p* = 0.02 ^†^	*p* = 0.08 ^‡^	*p* = 0.02 ^†^	*p* ≤ 0.01 ^†^	*p* = 0.36 ^‡^	*p* ≤ 0.01 ^†^	*p* ≤ 0.01 ^†^	*p* = 0.76 ^‡^
4.5 min + 2.5 min	12	114.56 ± 2.26 ^b^	29.89 ± 0.90 ^a^	11.93 ± 0.51 ^b^	99.99 ± 0.62 ^b^	294.75 ± 0.32 ^a^	887.54 ± 9.99 ^a^	10.65 ± 0.03 ^a^	3.26 ± 0.01 ^a^
4.5 min + 5 min	12	127.80 ± 2.26 ^a^	29.07 ± 0.90 ^a^	14.11 ± 0.51 ^a^	110.6 ± 0.62 ^a^	294.13 ± 0.32 ^a^	911.25 ± 9.99 ^a^	10.53 ± 0.03 ^b^	3.27 ± 0.01 ^a^
4.5 min + 7.5 min	12	107.90 ± 2.26 ^b^	26.40 ± 0.90 ^a^	11.27 ± 0.51 ^b^	95.69 ± 0.62 ^c^	294.15 ± 0.32 ^a^	741.97 ± 9.99 ^b^	10.30 ± 0.03 ^c^	3.26 ± 0.01 ^a^
Dataset average	36	117.93 ± 0.70	28.03 ± 0.28	13.38 ± 0.19	107.66 ± 0.23	293.93 ± 0.09	852.41 ± 5.42	10.12 ± 0.01	3.25 ± 0.01

Results are expressed as mean ± standard error. Values represented with different letters are statistically different at *p* ≤ 0.05; ^†^ significant factor in multifactor analysis; ^‡^ not significant factor in multifactor analysis. TPC—total phenolic content (mg 100 g^−1^); HCA—hydroxycinnamic acids (mg 100 g^−1^); FL—flavonols (mg 100 g^−1^); CT—condensed tannins (mg 100 g^−1^); Antioxidant activity—DPPH (µmol 100 g^−1^) and FRAP-(µmol 100 g^−1^); SSC—soluble solids content (°Brix). PEF—pulsed electric field (30 kV cm^−1^, 100 Hz); HPU—high-power ultrasound (amplitude 25%, pulse 50%).

**Table 4:** Mutual correlations of hurdle technology parameters on polyphenolic content, antioxidant capacities, SSC, and pH.

	Storage	PEF Exposure	HPU Exposure	TPC ^1^	HCA ^2^	FL ^3^	CT ^4^	DPPH ^5^	FRAP ^6^	SSC ^7^	pH
Storage	1	0	0	−0.07	−0.85 *	−0.3	0.37 *	−0.96 *	−0.19	0.03	−0.73 *
PEF exposure		1	0	−0.07	0.13	−0.24	−0.38 *	0.11	0.09	0.75 *	0.30 *
HPU exposure			1	−0.17	−0.05	−0.08	−0.19	0.08	−0.16	0.08	0.09
TPC				1	0.08	0.14	0.69 *	0	0.74 *	0.09	0.19
HCA					1	0.38 *	−0.40 *	0.90 *	0.18	0.08	0.68 *
FL						1	−0.05	0.35 *	−0.05	−0.19	0.16
CT							1	−0.46 *	0.54 *	−0.33	−0.43 *
DPPH								1	0.17	0.04	0.74 *
FRAP									1	0.25	0.26
SSC										1	0.28
pH											1

* Correlation is significant at the *p* ≤ 0.05. ^1^ TPC—total phenolic content (mg 100 g^−1^); ^2^ HCA—hydroxycinnamic acids (mg 100 g^−1^); ^3^ FL—flavonols (mg 100 g^−1^); ^4^ CT—condensed tannins (mg 100 g^−1^); ^5^ DPPH assay (µmol 100 g^−1^); ^6^ FRAP assay (µmol 100 g^−1^); ^7^ SSC—soluble solids content (°Brix). PEF—pulsed electric field (30 kV cm^−1^, 100 Hz); HPU—high-power ultrasound (amplitude 25%, pulse 50%).

**Table 5:** Optimal hurdle parameters for maximum content of polyphenols and antioxidant activity in the samples.

	TPC	HCA	FL	CT	DPPH	FRAP
Storage (Days)	0.0	0.0	0.0	0.0	0.0	7.0
PEF treatment (min)	1.5	1.5	4.5	1.5	2.7	3.0
HPU treatment (min)	2.5	7.5	5.8	3.2	7.5	7.5
Optimal quantity (mg 100 g^−1^)	125.81	35.56	17.36	116.72	301.22	958.82

TPC—total phenolic content (mg 100 g^−1^); HCA—hydroxycinnamic acids (mg 100 g^−1^); FL—flavonols (mg 100 g^−1^); CT—condensed tannins (mg 100 g^−1^); Antioxidant activity—DPPH (µmol 100 g^−1^) and FRAP (µmol 100 g^−1^).

## Data Availability

The data used to support the findings of this study can be made available by the corresponding author upon request.
